# Multifaceted, unique role of CD11c in leukocyte biology

**DOI:** 10.3389/fimmu.2025.1556992

**Published:** 2025-03-04

**Authors:** Lifei Hou, Sophia Koutsogiannaki, Koichi Yuki

**Affiliations:** ^1^ Cardiac Anesthesia Division, Department of Anesthesiology, Critical Care and Pain Medicine, Boston Children’s Hospital, Boston, MA, United States; ^2^ Department of Anaesthesia and Immunology, Harvard Medical School, Boston, MA, United States

**Keywords:** integrin, complement, CD11c, neutrophil, dendritic cell, T cell, B cell

## Abstract

CD11c is widely known as a dendritic cell surface marker but its non-dendritic cell expression profiles as well as its functional role have been gradually delineated. As a member of leukocyte-specific β2 integrin family, CD11c forms a heterodimer with CD18. CD11c/CD18 takes different conformations, which dictate its ligand binding. Here we reviewed CD11c current state of art, in comparison to its sister proteins CD11a, CD11b, and CD11d, illustrating its unique feature in leukocyte biology.

## Introduction

CD11c is largely known as a dendritic cell surface marker in immunology field (1). In fact, mice strains such as CD11c-Cre mice (2) and CD11c-diphtheria toxin receptor (DTR) transgenic mice (3, 4) have been widely used to study the function of DCs.

Over years, significant knowledge on CD11c has been accumulated, often investigated under different nomenclatures. Biologically, CD11c forms a heterodimeric adhesion molecule (CD11c/CD18) by coupling with CD18 (β2 subunit). CD11c/CD18 is named αXβ2 in the integrin field and belongs to leukocyte adhesion molecule β2 integrin family (5). It is also called complement receptor 4 (CR4) in the complement field as it binds to complement component iC3b (6). Furthermore, it has been increasingly recognized that CD11c is expressed beyond dendritic cells and plays a functional role rather than just a ‘surface marker’. For example, the presence of abundant CD11c-expressing blood neutrophils was recently considered as a potential biomarker for sepsis (7); CD11c- expressing B lymphocytes were considered to pivotally contribute to lupus pathology (8, 9). Thus, here we will review CD11c current state of the art.

## Protein structures and signaling pathways

**Figure 1:**
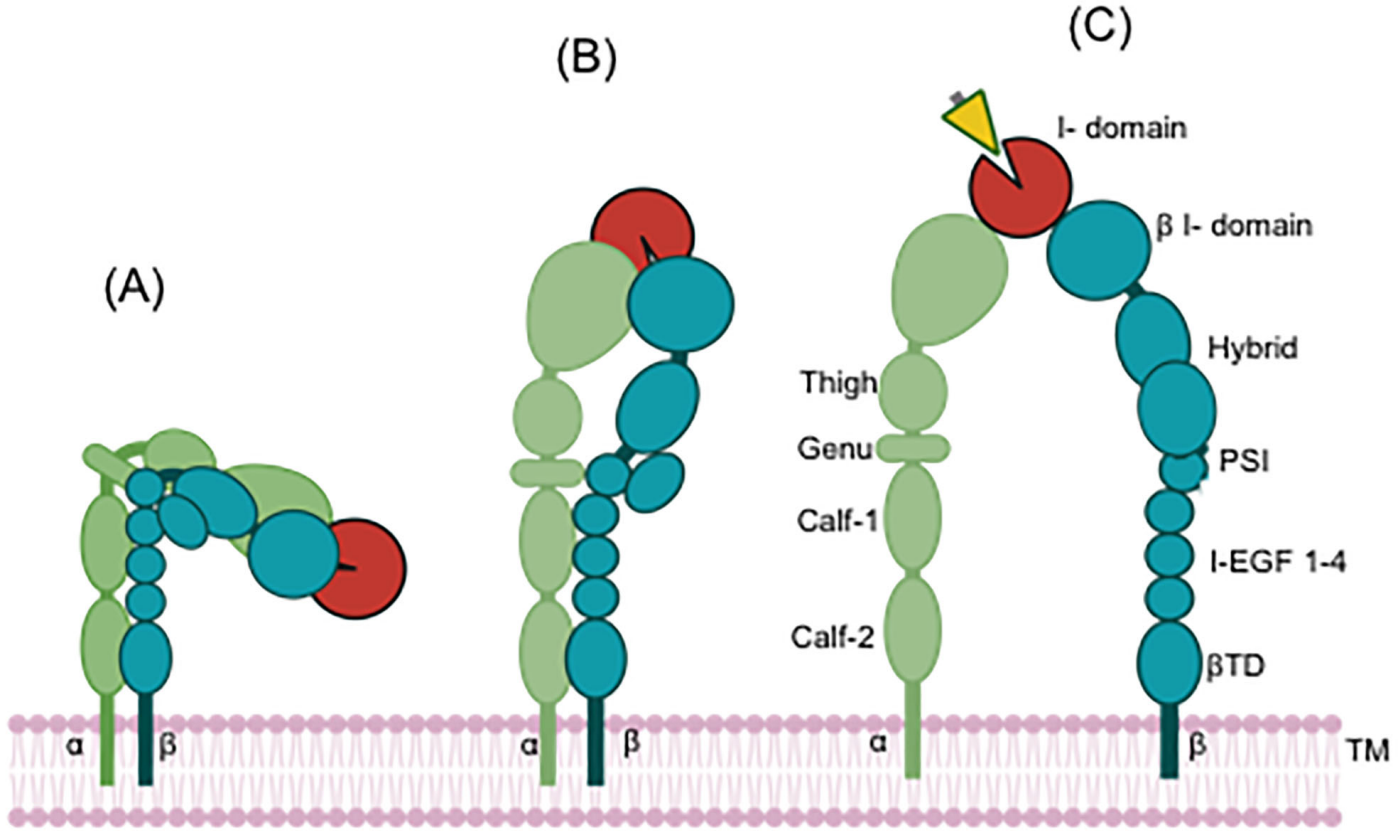
Conformational change of the I domain containing integrin. Red indicates the αI domain. Yellow indicates a ligand. **(A)** Closed conformation (inactive, resting status), **(B)** Closed-head conformation (Intermediate state), **(C)** Open-head conformation (active state). The ligand binds to integrin at open-head conformation. TM, transmembrane.

Integrins are heterodimeric adhesion molecules consisting of non-covalently associated α- and β-subunits and mediate cell-to-cell and cell-to-matrix interactions. 18 α- and 8 β-subunits have been identified so far, forming at least 24 distinct α/β heterodimers. CD11c (αX) was cloned by an integrin expert Dr. Springer’s group in 1990 (10). CD11c/CD18 (αXβ2) belongs to leukocyte-specific β2 integrin family, which consists of the four members; CD11a/CD18 (αLβ2), CD11b/CD18 (αMβ2, CR3), CD11c/CD18 (αXβ2, CR4) and CD11d/CD18 (αDβ2) (4). The conformational changes of integrin have been widely recognized by electron microscopy (EM), small angle X-ray scattering (SAXS), and X-ray crystallography studies (11–16). It largely takes three distinct conformations; 1) bent-closed (low affinity) where the extracellular domains of both α and β subunits are bent in the middle with the ligand binding domain being pointing toward the cell membrane, 2) extended-closed (intermediate affinity) where the extracellular domains of both α and β subunits are extended, but the ligand binding domain is not fully exposed, and 3) extended-open (high affinity) where the extracellular domains of both α and β subunits are extended, and the ligand binding domain is fully exposed to its ligands (17, 18) (**Figure 1**). Integrin in the extended-open confirmation is considered functionally active, binding to its ligands. The structure of CD11c/CD18 in both closed and open conformations was solved by X-ray crystallographic study (19, 20), supporting this scheme. A subset of integrin α subunits contains a sequence homologous to von Willebrand α domain, called the Inserted domain (I domain), which serves as a ligand binding domain (21). Thus, integrins are divided into the I domain integrin and the I-less integrin based on the sequencing of the top domain of α subunit. As β2 integrin belongs to the I domain integrin (i.e. α subunit has the I domain), ligands for CD11c/CD18 were sought mainly by using the αX I domain protein. Various molecules are reported to bind to the αX I domain including iC3b (5), intercellular adhesion molecule-1 (ICAM-1) (22), fibrinogen (23), and heparin (24) *in vitro*. Its binding to complement component iC3b is a reason to be named as CR4. The interaction of CD11c/CD18 with these ligands *in vivo* needs verification. Among the four β2 integrin members, CD11b shows the most similar characteristic as CD11b also binds to iC3b, ICAM-1, and fibrinogen (25). However, CD11b is inclined to bind more to positively charged species, while CD11c binds to strongly negatively charged species (6), suggesting that CD11b and CD11c bind to the same ligand, but at different site (26).

Integrins are in an inactive conformation at baseline. The activation of receptors such as G protein-coupled receptors (GPCRs) and chemokine receptors induces a cascade events, inducing the structural changes (**Figure 1**) and allowing the integrin to bind to its ligand (Inside-out signaling) (27). When integrin binds to its ligand, it undergoes cytoskeletal changes via focal adhesion molecules, leading to cell proliferation, survival, differentiation and migration. Regarding β2 signaling pathway, it was most studied in CD11a/CD18 (αLβ2) (28). In case of CD11c, phosphoinositide 3-kinase (PI3K)/Akt pathway is involved (29), but detailed molecular interactions need future investigation.

## The role of CD11c in various leukocyte types

### Neutrophils

The expression of CD11c in neutrophils has been sporadically reported in the literature. Rorvig et al. performed proteome profiling of human neutrophil granule subsets and showed that CD11c was detected in gelatinase granules (30). We also demonstrated that both murine and human neutrophils had significantly high intracellular CD11c expression, while they had limited CD11c expression on the cell surface by flow cytometry and fluorescence microscopy studies (29). We further performed granule separation (31) and confirmed that CD11c was highly expressed in the compartment corresponding to gelatinase granules, along with secretory granules (32).

**Figure 2:**
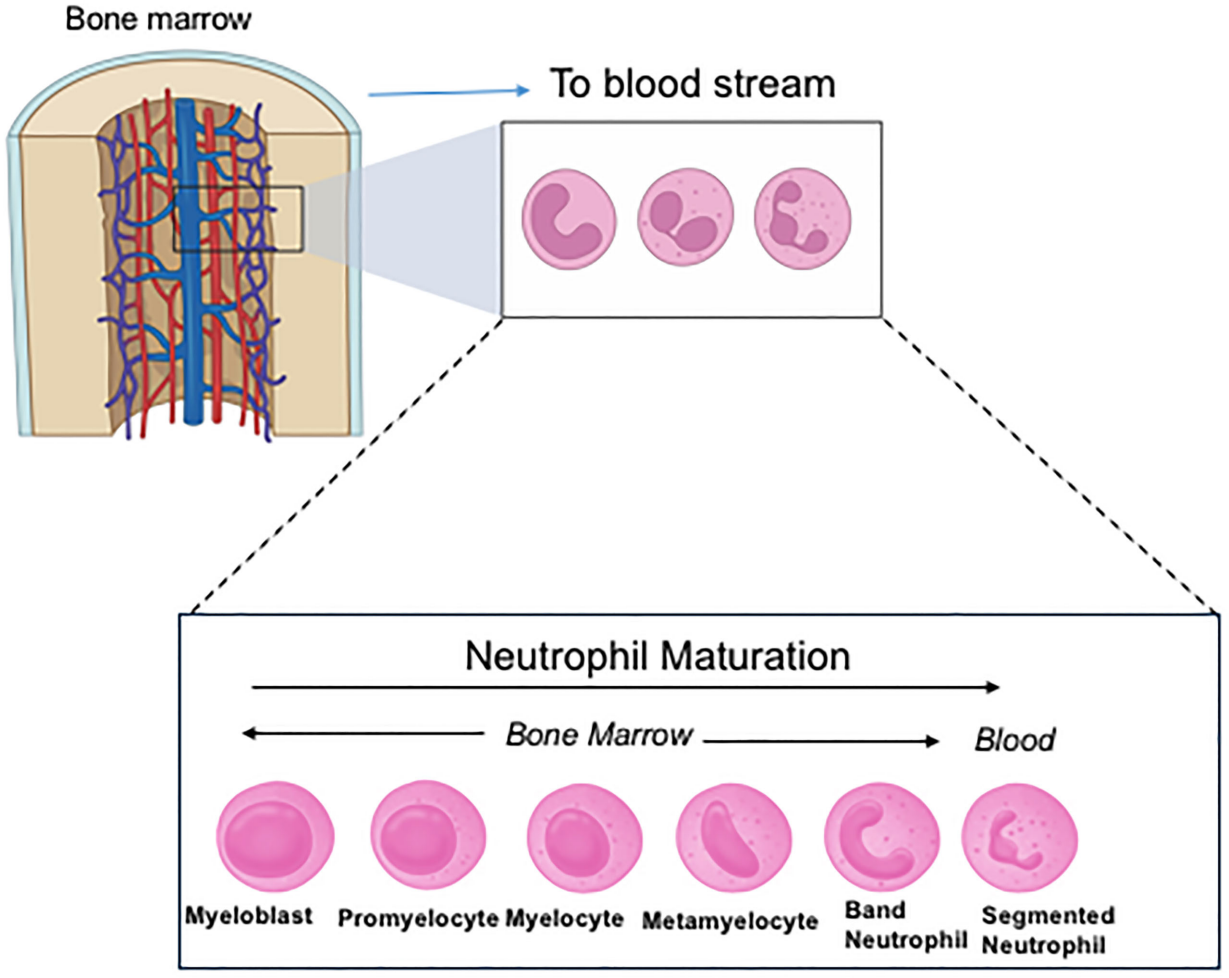
Neutrophil maturation and differentiation. In the figure, band neutrophils are considered immature neutrophils and segmented neutrophils are mature neutrophils. Neutrophils prior to band neutrophils are called pre-neutrophils. Mature neutrophils are primarily released into the peripheral blood. However, under stress and/or infection, immature neutrophils are also released into the circulation.

Unexpectedly we found that CD11c knockout (KO) mice had more immature neutrophils in the bone marrow (BM) compared to their wild-type (WT) counterpart (**Figure 2**) (29). In line with this finding, CD11c KO BM neutrophils showed less effector functions (phagocytosis, chemotaxis, reactive oxygen species (ROS) formation, neutrophil extracellular traps (NETs) formation) than WT BM neutrophils. While peripheral blood neutrophil counts were not different between WT and CD11c KO mice, neutrophils in the peripheral blood were less mature in CD11c KO mice compared to ones in WT mice (29). Although conventional DCs play a regulatory role in releasing neutrophils from the BM into their peripheral blood and their survival (33), mixed chimera (WT/CD11c KO) mice demonstrated less maturation of CD11c KO BM-derived neutrophils, supporting the idea that CD11c intrinsically regulates neutrophil maturation. This was further supported *in vitro* as CD11c deficient HL60 cells showed less neutrophil maturation/differentiation. The reduced maturation of CD11c KO BM neutrophils was driven by their exaggerated proliferation and apoptosis involving PI3K/Akt signaling pathway. We also created CD11c constitutive active knock-in mice (CD11c I334G mice) by locking the I domain in CD11c (αX) in the open (active) conformation (29). The mice showed significantly more mature neutrophils in the BM compared to WT mice, further demonstrating the critical role of CD11c in neutrophil maturation. So far, the functionality of integrins has been predominantly demonstrated when they are on the cell surface. The functionality of CD11c in the intracellular space is a novel finding. As the αI domain serves as a ligand binding domain, we examined a potential CD11c ligand in this process using proteomics. IQGAP1 (IQ motif containing GTPase activating protein 1) was considered as a potential ligand (29). IQGAP1 deleted HL60 cells also demonstrated less neutrophil maturation, consistent with the result of CD11c deleted HL60 cells. IQGAP1 KO mice also showed less mature BM neutrophils, consistent with the phenotype of CD11c KO mice (32).

In addition to neutrophil maturation, CD11c also independently affected reactive oxygen species (ROS) formation. Mature CD11c KO BM neutrophils showed significantly less ROS formation compared to mature WT BM neutrophils upon phorbol 12-myristate 13-acetate (PMA) stimulation (29). Similarly, mature CDI334G KI BM neutrophils showed significantly more ROS formation than mature WT BM neutrophils. While PMA induces ROS formation by activating protein kinase C and nicotinamide adenine dinucleotide phosphate (NADPH) oxidase, how CD11c affects this mechanism remains to be determined. In contrast to ROS formation, phagocytosis was not different between mature WT and CD11c KO BM neutrophils, suggesting that CD11c specifically regulates ROS formation among neutrophil effector functions.

CD11c is expressed on the cell surface of neutrophils, though its expression is significantly less than in the intracellular space (29). CD11c cell surface expression is upregulated on aged neutrophils (34, 35). Aged neutrophils have more active effector functions in acute infection associated with more β2 integrin activation, probed by ICAM-1 binding (36). However, the involvement of CD11c in the augmented effector function of aged neutrophils has not been clarified yet. In addition, the abundance of CD11c expressing neutrophils has been correlated with the sepsis severity in the patient (7). All of these experimental findings above and patient data suggest that CD11c might serve as a target to regulate the neutrophil maturation in various disease conditions including sepsis.

### Monocytes/macrophages

CD11c is also expressed on monocytes. CD11c is highly expressed on intermediate and non-classical monocytes, while its expression on classical monocytes is limited in humans. Compared to CD11b expression level, CD11c expression is approximately 1/7 on monocytes as a whole (37). CD11c^+^ monocytes are considered as activated monocytes, associated with an increase in endocytic activity (38). Sandor et al. found *in vitro* that CD11b was predominantly involved in iC3b-mediated phagocytosis (39), while CD11c was more adhesive to fibrinogen over CD11b (37).

A subset of macrophages also expresses CD11c. Alveolar macrophages express CD11c, while interstitial macrophages show its limited expression (40). In the study on salivary gland macrophages, CD11c^-^ macrophages are derived from embryonic progenitors, while CD11c^+^ macrophages are from the bone marrow derived progenitors (41). Whether or not CD11c has a functional role in macrophages is yet to be determined.

### Dendritic cells

**Figure 3:**
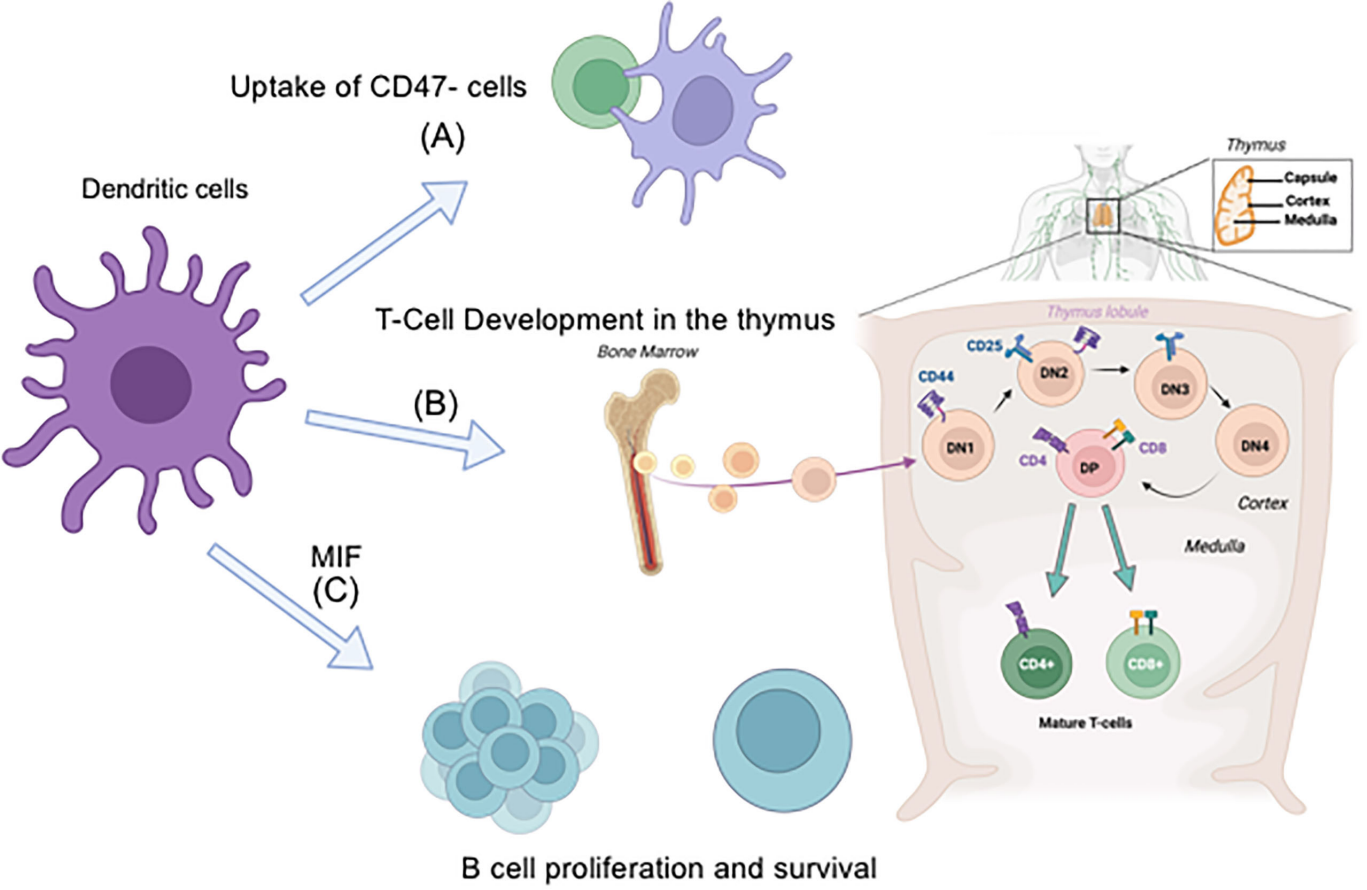
The role of CD11c on DCs in DC functions, T cell development in the thymus, and B cell proliferation and survival. **(A)** CD11c is involved in the uptake of CD47^-^ cells by DCs, **(B)** Progenitor cells will be mobilized from the bone marrow to the thymus then T cells are developed in the thymus. DCs are involved in negative selection. **(C)** Macrophage inhibitory factor (MIF) is produced by DCs. MIF binds to B cells and induce their proliferation/survival.

CD11c is widely used as a defining marker for DCs for a long time (42), but its functional role has been less explored. CD47 is a cell surface protein abundantly expressed on most cell types and known as a “marker of self”. CD47 engagement to the Signal regulatory protein alpha (SIRPα) receptor transmits a “don’t eat me” signal to DCs and macrophages to prevents their engulfment (43). When CD47 is missing from erythrocytes in circulation, they are rapidly cleared by macrophages (44). Splenic CD4^+^ DCs also express SIRPα and directly interact with circulating erythrocytes in the marginal zone. This causes strong CD4^+^ DCs activation, accompanied by the migration and accumulation of these DCs to the T cell zones of the splenic white pup, where they activate CD4^+^ T cells (45). Using CD11c KO mice, Wu et al. showed that CD11c was critically involved in the binding to and uptake of CD47-deficient cells by DCs (42) (**Figure 3**). Although they explored the possible involvement of several reported CD11c ligands including iC3b in this biological event, the ligand remains to be determined as of now. Of note, CD11c deficiency did not affect the number of conventional DC1 (cDC1, MHC-II^+^XCR1^+^CD8a^+^), cDC2 (MHC-II^+^XCR1^-^CD8a^-^SIRP1^+^CD11b^+^), and plasmacytoid DC (pDC, PDCA^+^CD11b^-^Ly6C^+^) subsets (42, 46). DCs serve as antigen presenting cells (APCs). DCs in CD18 KO mice, which lacks all functional β2 integrins, have no defect in antigen presentation (47). Thus, it is unlikely that active CD11c is functionally required for DC’s antigen presentation.

Depletion of CD11c+ cells in the CD11c-DTR model drives the expansion of unique CD64^+^ Ly6C monocyte population in the blood, which upregulates TLR signaling apparatus and is more poised to produce proinflammatory cytokines (48). These monocytes have been reported in tissues (49). Because monocytosis was also reported in zinc finger and BTB domain containing 46 (Btbd4, zDC) mediated DC deletion mice (50), the effect of CD11c-DTR model on monocytes is likely driven by DCs, not CD11c.

### NK cells

A subset of NK cells also express CD11c on their cell surface. The study by Aranami et al. showed that 20-80% of NK cells from healthy subjects were CD11c positive (51). Furthermore, IL-15 induced CD11c expression on the cell surface of NK cells *in vitro*. Similar to monocytes/macrophages, the functional role of CD11c in NK cells is limitedly studied, waiting for future insight.

### T cells

Although not frequent, CD11c expression has been reported on the cell surface of a subset of CD8 T cells (52). CD11c^+^ CD8 T cells suppress autoreactive CD4 T cells (53). CD11c^+^ CD8 T cells also show cytotoxicity, stronger than CD11c^-^ CD8 T cells (54). As anti-CD11c antibody inhibits cytotoxicity, CD11c on the cell surface may facilitate conjugation (55). In addition, a subset of γδT cells expresses CD11c. CD11c+ γδT cells secrete more interferon gamma (IFN-γ) secretion upon activation than CD11c^-^ γδT cells, suggesting that they have a more robust effector function (56).

While the majority of T cells does not express CD11c, peripheral CD4 and CD8 T cell counts are significantly lower in CD11c KO mice than in WT mice in a steady state, suggesting that CD11c can regulate T cells (46). The thymus is the primary lymphoid organ that supports T cell development via three major stages: Double negative (DN), double positive (DP), and single positive (SP). During these stages, developing lymphocytes undergo a dynamic relocation and give rise to naïve T cells, which are released to the periphery (57, 58). CD11c KO mice showed smaller-sized thymus with the loss of cellularity, which was accompanied by a significantly less number of DP, CD4 SP, and CD8 SP cells. However, no difference in the number of DN cells between WT and CD11c KO mice was observed (46). The analysis of CD4 and CD8 SP population showed that they were skewed toward more mature population, indicating that immature CD4 and CD8 SP population was more affected in the thymus of CD11c KO mice.

During the development of T cells in the thymus, thymic epithelial cells (TECs) and DCs are involved in selection. Cortical TECs (cTECs) are involved in thymocyte positive selection, and medullary TECs (mTECs) and DCs are involved in negative selection (59, 60) (**Figure 3**). While TECs and developing T cells did not show any CD11c expression, DCs showed robust CD11c expression. The chimera of irradiated CD11c KO mice transplanted with WT donor BM cells (WT/CD11c KO chimera) had thymocyte number comparable to those of WT/WT chimera. Thus, it is possible that the effect of CD11c on T cell developing in the thymus is via DCs. Whether CD11c on DCs affects the phenotype of developing T cells via direct interaction or indirectly regulates by affecting surrounding milieu needs to be determined.

### B cells

CD11c expression is also reported in a subset of B cells in both mice and humans. The presence of CD11c^+^ B cells is well described in autoimmune diseases including rheumatoid arthritis (61), Sjogren’s syndrome (62), multiple sclerosis (63), and systemic lupus erythematosus (64). In addition, CD11c^+^ B cells are also reported in healthy individuals in an increasing frequency together with age (65). While CD11c expression is not seen in the cell surface of B cells in younger mice (66), CD11c^+^ B cells also accumulate with age in mice, which was initially named “age-associated B cells (ABC) (61). Golinski et al. studied the presence of CD11c^+^ B cells in transitional (CD19^+^CD24^+^CD38^+^), naïve (CD19^+^IgD^+^CD27^-^), memory B cells (CD19^+^CD27^+^) and plasmablasts (CD19^+^CD24^-^CD38^+^), most frequently seen in memory cells (65). They also found that CD11c^-^ B cells induced CD11c expression upon B cell receptor (BCR) stimulation. ABC was later named atypical B cells as they were considered part of an alternative lineage of B cells involved in responses to vaccination and infection (67). In fact, CD11c expression was considered a marker of atypical B cells based on single cell RNA sequencing (scRNA seq) analysis data and flow cytometry data (67, 68). While overall CD11c^+^ B cells seem to be associated with B cell activation, the functional role of cell surface CD11c in B cells remains to be determined. Interestingly, CD11c-DTR system depleted activated B cells including germinal center B cells (69). Thus, this system can be used to study atypical B cells. At the level of transcriptional regulation, ABC could also be characterized by the expression of the transcription factor T-bet. Indeed, T bet defines this B cell subset, which also expresses several other characteristic cell surface markers including CD11c, CD11b, and CD73 (70). Thus, it’s reasonable to hypothesize that CD11c expression on the B cell could be regulated by transcription factor T-bet.

CD11c cell surface expression is absent on circulating B cells in younger mice. However, B cell count is significantly lower in CD11c KO mice compared to WT mice (66). Particularly the number of CD11c KO recirculating and mature B cells is significantly lower compared to that of WT. Furthermore, CD11c KO B cells are associated with exaggerated proliferation and apoptosis. The analysis of mixed chimera mice showed that the regulation of B cell proliferation and apoptosis was non-intrinsically driven. CD11c KO DCs produced less macrophage migration inhibitory factor (MIF). CD74 is a receptor for MIF (71). The binding of MIF to CD74 on B cells activates PI3K/Akt pathway, regulating B cell proliferation and survival (72, 73) (**Figure 3**). DCs potentially regulate B cell number via CD11c. This needs further experimental clarification.

Overall, CD11c seems to affect B cells directly or indirectly. However, it is still elusive about the definite role of CD11c in the generation, accumulation, and effector functions of B cells. More experiments are needed in this regard.

### Bone marrow leukocytes

In addition to its expression on peripheral leukocytes, CD11c is also expressed on the short-term hematopoietic stem cells (ST-HSCs) and multipotent progenitor cells (MPPs). The lack of CD11c expression on these cells is associated with a significant increase in their proliferation and apoptosis under stress such as sepsis and bone marrow transplantation (74). CD11c KO mice show a significant loss of HSPCs under stress, indicating the critical role of CD11c in HSPCs in disease process (74).

In this section, we reviewed the involvement of CD11c in various leukocytes. The summary of CD11c expression profiles per each cell type is summarized in **Table 1**.

**Table 1:** CD11c expression profiles in immune cells.

	Intracellular expression	Cell surface expression
Neutrophils	+	+
Monocytes/Macrophages	ND	Activated monocyte and alveolar macrophage
Dendritic cells	+	+
NK cells	ND	A subset of NK cells
T cells	–	–
B cells	–	A subset of B cells (aged B cells)
LT-HSCs	–	–
ST-HSCs	–	+
MPPs	ND	+
CLPs	–	–
CMPs	ND	+
LSKs	ND	+

ND, not determined; LT-HSC, long-term hematopoietic stem cell; ST-HSC, short-term hematopoietic stem cell; MPP, multipotent progenitor cell; CLP, common lymphoid progenitor cell; CMP, common myeloid progenitor cell; LSK, Lin-Sca1+c-Kit+ population of the bone marrow.

## The comparison with other β2 integrin members

CD11a (αL), CD11b (αM), CD11c (αX), and CD11d (αD) are sister proteins and on chromosome 8. Evolutionally, CD11a and CD11b exist first. CD11b and CD11c are predicted to arise from gene duplication event (75). Then, CD11d appears last (76). From homology standpoint, the identity of the ligand binding domain and the β propeller domain between CD11b and CD11c is the highest (~77%) (42). Each β2 integrin member is expected to play a unique role. It is also important to understand if there is a significant redundancy in each member’s function.

### CD11a

CD11a is ubiquitously expressed on peripheral leukocytes and plays a major role in leukocyte functions, ranging from leukocyte adhesion to immunological synapse formation (77, 78). The major ligand for CD11a/CD18 is ICAM-1 (79, 80).

CD11a is not required for neutrophil differentiation and maturation (29), but CD11a significantly affects neutrophil extravasation (81). CD11a deficiency is also associated with neutrophilia (82, 83). This is likely a secondary phenotype due to a reduced egress of neutrophils from the peripheral blood to other compartments in CD11a KO mice, which induces higher circulating IL-23, IL-17, and granulocyte colony-stimulating factor (G-CSF) levels (84).

In DCs, CD11a activity state is kept in a low affinity state by re-localizing cytohesin-1, which is a molecule to interact with CD11a/CD18 from the plasma membrane to the cytosol (85). The activation of CD11a on DCs causes a prolonged contact between DCs and naïve T cells, which inversely correlates with T cell activation and antigen-specific T cell proliferation (86).

NK cells express a number of activating and inhibitory receptors on their cell surfaces to recognize stress ligands as well as MHC class I (87). CD11a is one of the activating receptors and serves as a major adhesion molecule on NK cells. For example, the binding to ICAM-1 on tumor cells can lead to the conjugation between NK cells and tumor cells, which results in the reorganization of cytoskeletal structures and lytic granule polarization within NK cells. CD11a deficiency impairs NK cell-mediated cytolysis (88, 89).

CD11a is also important for immunological synapse formation. T cell receptor (TCR) aggregates into a central supramolecular activation clusters (cSMACs) surrounded by a peripheral ring (pSMACs) of CD11a to form an immunological synapse between T cells and APCs such as DCs (90, 91). Thus, in contrast to CD11a on DCs, activated CD11a on T cells helps to stabilize an interaction between T cells and DCs and acts as a co-stimulator for T cell activation (92). Instead, B cells can form a functional synapse without CD11a when the avidity of B cells for the antigen exceeds a certain threshold (93). However, CD11a (on B cell) -ICAM-1 (on APC) interaction lowers the threshold of B cell activation by facilitating B cell activation and synapse formation. In addition, homotypic aggregation of B cells via CD11a-ICAM-1 regulates IgE synthesis by modulating C epsilon germ-line transcription (94).

CD11a is also expressed on all of long-term hematopoietic stem cells (LT-HSCs), ST-HSCs, MPPs, CLPs and CMPs (95). While CD11a deficiency enhanced HSPCs activity under lipopolysaccharide (LPS) stimulation, the mixed chimera (WT/CD11a KO) analysis did not support that this was cell-intrinsically driven. Instead this may be driven by IL-27 as its production was attenuated in CD11a KO mice (95). IL-27 is involved in the promotion of expansion and differentiation of HSCs in the setting of emergency myelopoiesis (96).

Overall CD11a shows a very different functionality on leukocytes compared to CD11c.

### CD11b

CD11b is mainly expressed on innate immune cells, but also on a subset of adaptive immune cells, as in the case of CD11c.

CD11b is highly expressed on neutrophils and monocytes and plays a major role in their recruitment, phagocytosis and cell death (97). While CD11b has the highest homology to CD11c and share the same ligands including iC3b, ICAM-1, and fibrinogen, CD11b is not required for neutrophil maturation and differentiation (29).

DCs also express CD11b. Similar to CD11a, CD11b on DCs is kept inactive. The presence of active CD11b on DCs inhibits full T cell activation (47).

A subset of NK cells expresses CD11b. CD11b has been considered as a marker of NK cell maturation (98–100). However, it has been reported that CD11b KO NK cells are more activated and have more cytolytic capability (101). Thus, the role of CD11b in NK cells needs to be studied more in depth.

CD11b is expressed on a subset of T cells including CD8 T cells and γδT cells (102). CD11b expression on CD8 T cells have been associated with acquisition of cytotoxic capacity (103).

CD11b is also expressed on a subset of B cells. CD11b plays a role in the maintenance of autoreactive B cell tolerance by providing negative regulation on BCR-mediated signaling (104).

BM cells in CD11b KO mice behave quite differently compared to CD11c KO mice. Under LPS stimulation, the number of CD11b KO HSPCs increased similarly to WT, CD11a KO and CD11d KO HSPCs, while CD11c KO mice showed their significant decrease (74). Interestingly, CD11b KO BM had higher CMP number compared to WT and other β2 integrin member KO mice (74). The underlying mechanism has not been delineated yet.

### CD11d

The reported ligands for CD11d/CD18 include ICAM-3 (105), vascular cell adhesion molecule 1 (VCAM-1) (106), and 2-(ω-carboxyethyl)pyrrole (CEP) (107), which are quite different from other β2 integrin members.

CD11d expression is reported mainly on myeloid cells (108). CD11d deficient neutrophils show reduced cell death and increased phagocytosis (109).

CD11d is expressed on foam cells, a type of macrophages filled with lipids (105). CD11d in foam cells promotes their retention in vascular lesions and development of atherosclerosis (110).

While CD11d is reported on a subset of NK cells, γδT cells, and B cells (111). However, CD11d KO BM cells did not show any significant difference compared to WT BM cells (74).

### Sepsis as a model to compare the function of the four β2 integrin members

As reviewed above, each β2 integrin member demonstrates redundancy as well as uniqueness. To illustrate the role of each β2 integrin member *in vivo*, we reviewed the data from the experimental sepsis as an example. The critical role of β2 integrin in infection has been well recognized by a genetic disorder leukocyte adhesion deficiency type I caused by functional defect β2 integrin, characterized by recurrent infections, impaired pus formation, and sepsis (112). In line, CD18 (β2) KO mice showed significantly higher mortality in polymicrobial abdominal sepsis induced by cecal ligation and puncture surgery compared to WT mice (109). From the mortality standpoint, CD11a KO, CD11b KO, and CD11c KO mice showed higher mortality compared to their corresponding WT mice (74, 82, 113, 114), similar to what was observed in β2 KO mice. However, the analysis of these three KO mice in the sepsis model revealed quite different immune cell behaviors despite a similar outcome. CD11a KO mice showed a significantly reduced number of migrated neutrophils to the peritoneal cavity, which was associated with more bacterial loads (114). However, their neutrophils had competent phagocytosis. In contrast, CD11b KO mice had normal migration of neutrophils to the peritoneal cavity, but showed impaired neutrophil phagocytosis with more bacterial loads (114). CD11c KO mice showed significantly impaired neutrophil maturation, which was associated with a reduction in all the neutrophil effector functions. In contrast, CD11d KO mice showed better sepsis survival compared to WT mice (109). As CD11d KO neutrophils showed significantly reduced apoptosis with more available neutrophils compared to WT mice, they showed less bacterial loads. However, CD11d expression in neutrophils is limited compared to CD11a, CD11b and CD11c.

## Future research direction

While CD11c has a very unique feature in leukocyte functions including neutrophil maturation, ROS formation, and DC uptake of CD47^-^ cells, but the role of cell surface CD11c in a subset of B cells, NK cells, and T cells, for example, is not well explored. As CD11c cell surface expression on those cells seem to overlap with CD11b, and CD11d, a clear understanding of an individual player’s role on their cell surface is critical. While lots of research need to be done, CD11c may serve as an exciting target.

Since CD11c is involved in the maturation and/or effector functions of several myeloid-derived cells including neutrophils, DCs, and monocytes, manipulating CD11c would serve as a potential strategy to intervene diseases involving these cells, which include tumor and autoimmune diseases other than acute infectious diseases. For example, myeloid-derived suppressor cells (MDSCs) accumulate in the tumor microenvironment and contribute to the resistance to cancer therapy. CD11b agonist has already been reported as a strategy to re-program the MDSCs to overcome the suppressive tumor microenvironment (115, 116). Neutrophils play a major role in breast cancer with anti-tumor (N1) and pro-tumor (N2) neutrophils (117). In this regard, CD11c activation could be a potential strategy to enhance neutrophil functions. While limited literature is available regarding the role of CD11c in tumor, future investigation is needed. Since CD11c controls the neutrophil ROS generation and NETs formation, CD11c antagonist should be explored as a potential therapeutic for treating lupus, where NETs play a major role and the other nuclear antigens are released during NETs formation (118–120). Targeting CD11c could also suppress CD11c-positive pathogenic B cells, which provide an additional rationale to design CD11c-based therapeutic for lupus.
